# 3D cryo-EM imaging of bacterial flagella: Novel structural and mechanistic insights into cell motility

**DOI:** 10.1016/j.jbc.2022.102105

**Published:** 2022-06-06

**Authors:** Sonia Mondino, Fabiana San Martin, Alejandro Buschiazzo

**Affiliations:** 1 Laboratory of Molecular & Structural Microbiology, Institut Pasteur de Montevideo, Montevideo, Uruguay; 2 Integrative Microbiology of Zoonotic Agents IMiZA Unit, Joint International Unit, Institut Pasteur/Institut Pasteur de Montevideo, Paris/Montevideo, France/Uruguay; 3 Microbiology Department, Institut Pasteur, Paris, France

**Keywords:** allosteric regulation, cell motility, molecular motor, protein self-assembly, protein–protein interaction, proton motive force, proton transport, Spirochetes, structural biology, CCW, counter-clockwise, cryo-ET, cryo–electron tomography, CW, clockwise, PG, peptidoglycan, SPA, single-particle analysis, T3SS, type-three secretion system

## Abstract

Bacterial flagella are nanomachines that enable cells to move at high speeds. Comprising 25 and more different types of proteins, the flagellum is a large supramolecular assembly organized into three widely conserved substructures: a basal body including the rotary motor, a connecting hook, and a long filament. The whole flagellum from *Escherichia coli* weighs ∼20 MDa, without considering its filament portion, which is by itself a ∼1.6 GDa structure arranged as a multimer of ∼30,000 flagellin protomers. Breakthroughs regarding flagellar structure and function have been achieved in the last few years, mainly because of the revolutionary improvements in 3D cryo-EM methods. This review discusses novel structures and mechanistic insights derived from such high-resolution studies, advancing our understanding of each one of the three major flagellar segments. The rotation mechanism of the motor has been unveiled with unprecedented detail, showing a two-cogwheel machine propelled by a Brownian ratchet device. In addition, by imaging the flagellin-like protomers that make up the hook in its native bent configuration, their unexpected conformational plasticity challenges the paradigm of a two-state conformational rearrangement mechanism for flagellin-fold proteins. Finally, imaging of the filaments of periplasmic flagella, which endow Spirochete bacteria with their singular motility style, uncovered a strikingly asymmetric protein sheath that coats the flagellin core, challenging the view of filaments as simple homopolymeric structures that work as freely whirling whips. Further research will shed more light on the functional details of this amazing nanomachine, but our current understanding has definitely come a long way.

Flagella are large supramolecular assemblies that work as nanomachines in bacteria, enabling the cells to swim in liquid environments at high speeds. In this way, bacteria move at ∼30 to 150 μm/s (1, 2, 3), reaching even higher velocities in some cases (4). A 1 μm sized bacterium swimming at 100 μm/s (∼36 cm/h) advances ∼100 times its own body length per second, which is roughly equivalent to a 1.7 m sized person swimming at >600 km/h. As a reference, normal swimming speeds for an average human being are in the range of 3 km/h, and the fastest professional swimmers can attain ∼10 km/h. Bacterial flagella use energy very efficiently to turn on their motor and make it rotate at rates on the order of ∼100 to 270 Hz (6000–16,000 revolutions per minute) (5, 6) or even up to sixfold faster in extreme cases (7). Beyond motility, flagella mediate several processes, some of them relevant in host–pathogen interactions, including (i) biofilm formation and bacterial adherence to substrates, like host cells and tissues (8, 9); (ii) the secretion of virulence-associated proteins *via* the flagellar export apparatus (10); and (iii) immunomodulation and evasion from host immune response (11). The structure and biogenesis of bacterial flagella, and the diverse array of biological roles they are involved in, have been the subject of excellent reviews (12, 13, 14), hence not further discussed in this article.

Instead, this review is focused on a few selected motility functions that flagella are known to be engaged in, which have received recent attention because of the radical new mechanistic insights that have been uncovered. Double-membrane Gram-negative Enterobacteria, such as *Salmonella enterica* and *Escherichia coli*, have served over time as preferred models to study the structure and function of bacterial flagella, largely contributing to current bacterial motility paradigms (15). The bacterial flagellum is organized into three basic substructures: (i) *the basal body*, which anchors the flagellum into the cell membrane and comprises a protein export apparatus, a rotary motor connected to a central drive shaft or rod, and bearing structures; (ii) *the hook*, which is directly joined to the rod and acts as a rotating universal joint coupling the motor to the filament along disparate axes; and (iii) *the filament*, an extremely long appendage that operates as a propeller generating thrust (16) (Fig. 1). The entire flagellum is built with >25 different types of proteins, some of them present as single components, yet others repeated in tens of thousands of copies. In *E. coli*, each flagellum weighs ∼20 MDa, without considering the filament, which is on its own a ∼1.6 GDa polymer (comprising ∼30,000 flagellin protomers). In Gram-positive bacteria, most of the flagellar appendage is extracellular, with only part of the basal body including cytoplasmic and transmembrane structures (17). A similar cellular topology is exhibited by most Gram-negative species, with their basal body extending through the periplasm, the tip of their rod typically traversing the outer membrane, and their hook and filament exposed toward the extracellular milieu (18).

**Figure 1:**
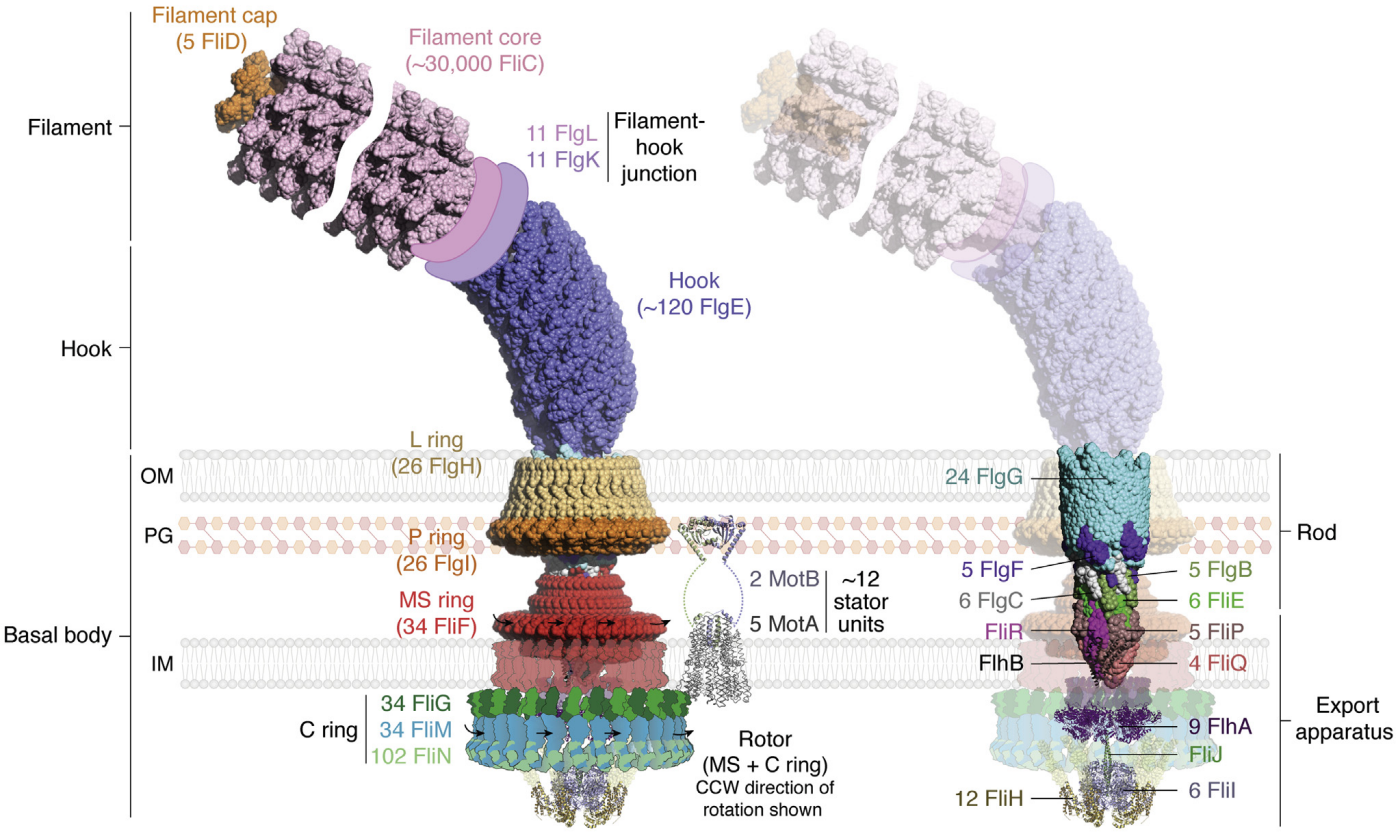
**Molecular architecture of the bacterial flagellum.** Cartoon representation of a complete bacterial flagellum, juxtaposing high-resolution experimental structures of subassemblies, with proper interconnecting geometries. All the components are drawn to scale. The *left panel* shows solid molecular surfaces for most of the appendage. The *right panel* is identical, except that outermost solvent-exposed protein complexes are rendered semitransparent, uncovering the inner composition of the protein export apparatus and rod. There are no structures of assembled hook-associated proteins FlgL and FlgK (only structures of individual protomers); they are thus represented by two schematic rings at the hook–filament junction. There is no deposited PDB for the FliG–FliM–FliN C ring assembly (only structures of protomers or subregion complexes), corresponding to the fitted model within the available cryo-ET reconstruction volumes (41), hence a schematic illustration is shown (adapted from Ref. (41)). PDB IDs used to build this drawing: 6SIH (FliD filament–capping complex from *Campylobacter jejuni*); 1UCU (FliC flagellin filament from *Salmonella enterica*); 6K3I (FlgE hook from *S. enterica*); 7CGO (FlgH L ring; FlgI P ring; FliF MS ring; the rod components FlgG, FlgF, FlgC, FlgB, and FliE; the export apparatus components FliP, FliQ, and FliR; all from *S. enterica*; note that the transmembrane and C ring–connecting regions of the MS ring were not well resolved in the original cryo-EM maps (110), hence depicted as a semitransparent *red surface*); 6S3L (FlhB, from the complex with FliPQR from *Vibrio mimicus*); 7AMY (FlhA ring, cytoplasmic region, from *Vibrioparahaemolyticus*; the FlhA transmembrane region is represented as a semitransparent *violet* volume, since it could not be solved (111)); 5B0O (part of the ATPase complex corresponding to the FliH–FliI complex from *S. enterica*; ATP-synthase 6OQR was used as template to generate the FliH–FliI hexameric assembly; FliH N-terminal extension arms are shown semitransparent going up toward the C ring, they were modeled with AlphaFold2 with high reliability); 3AJW (FliJ subunit of the ATPase complex from *S. enterica*); 6YKM (transmembrane portion of the MotA–MotB complex from *Campylobacter jejuni*); and 2ZVY (MotB PG-binding OmpA-like domain from *S. enterica*). ET, electron tomography; IM, inner membrane; OM, outer membrane; PDB, Protein Data Bank; PG, peptidoglycan.

The flagellar apparatus is a fascinating illustration of evolutionary variation, sometimes leading to completely new functions based on conserved shared structures. A most extreme example of such a functional drift is the evolution of type-three secretion systems (T3SSs or injectisomes) from the flagellar ancestor (19). The T3SS is a multiprotein assembly that has conserved many components of the flagellar protein export apparatus. Ultimately specializing in protein secretion, T3SSs lost several protein components, and with them, the abilities to rotate and drive motility altogether, while also evolving several unique elements to attain its exquisite protein secretion/injection capacities. We shall not address T3SS function and structural variations with further detail here, instead pointing the reader to several reviews on the subject (13, 20, 21, 22). Less drastic architectural variations of rotating flagella are also observed, which can sometimes radically modify the locomotion mechanism. The hooks and filaments of several Gram-negative bacteria, such as *Vibrio*, *Helicobacter*, *Brucella*, and related genera, protrude extracellularly, yet are fully surrounded by the uninterrupted sheath of the outer membrane (23).

A more extreme modification is observed in the entire Spirochete phylum, where flagella, known in this group as endoflagella, are entirely confined within the periplasm, with the filament helically wrapped around the cell body (23). Although such endoflagella are very similar to typical exoflagella regarding their architecture and protein composition, the fact that they evolved as an entirely periplasmic machine is driving an ongoing shift of the accepted bacterial motility paradigm. Even though further research is needed to fully elucidate the endoflagellar motility mechanism, it seems clear that their role involves a form of “dragging” the bacterial cell body and influencing cell morphology (24, 25). Accordingly, Spirochetes possess flagellar motors that can exert the highest torques so far observed in bacteria (26), with the rest of the assembly particularly adapted to endure extreme rotation regimes within such a restricted volume of the cell. Torque is a vectorial quantity that represents the capability of a force to produce change in the rotational motion of a body. Analogous to a linear force’s push and pull, torque can be thought of as a twist around a specific axis. It is the cross product of two vectors: (i) the distance of a rotating body—or a defined point in that rigid body—to the axis of rotation and (ii) the force applied to that body for it to rotate. Hence, the magnitudes of torque depend not only on the force applied but also on the length (radius) of the rotating object and the angle between them. These concepts will be important in appreciating the different means by which evolution has modulated higher or lower torques in different bacterial species.

The ongoing revolutionary progress of three-dimensional cryo-EM and cryo–electron tomography (cryo-ET) approaches (27, 28) has recently contributed to breakthrough observations of flagellar structures and their connection to the molecular mechanisms that underlie bacterial locomotion. In this review, we discuss major advances in structure–function relationships concerning the flagellar motor, hook, and filament, and future research directions that might answer some of the remaining open questions. We also highlight unique variations in Spirochete flagella, as a model to showcase how evolution uses an ancestral common structure to attain distinct mechanisms of translational motility in bacteria.

## The flagellar motor: A nanomachine driven by a Brownian ratchet mechanism

The flagellar basal body harbors the motor device that drives flagellar rotation, while also including additional protein components that play key roles (Fig. 1): (i) a multiprotein export apparatus traversing the cell membrane and intruding into the cytoplasm, which ensures secretion of axial proteins during flagellar biogenesis; (ii) a set of stacked protein rings (C and MS rings), which constitute the moving rotor of the motor, and its switch complex that allows the direction of rotation to be inverted; (iii) several proteinaceous ring bearings (the P and L rings), isolating moving parts from fixed wall elements; and (iv) a hollow central shaft or rod, which connects the rotor to the hook. The rotor associates dynamically to the membrane-embedded and peptidoglycan (PG)-bound MotA–MotB stator units, which constitute the motor-force generators (29) (Fig. 1).

In a general sense, motors are engines that perform mechanical work by using a two-component arrangement: a static part (stator) is fixed to a reference framework, whereas a moving part (rotor) rotates with respect to the stator. Some form of chemical and/or electrical input energy is needed to drive rotation unidirectionally. Brownian (random) back and forth small rotations because of thermal energy in the system would not produce effective work, as for the purpose of cell movement. For any form of energy to be useful at the nanometric scale of protein parts, such energy input must produce allosteric changes in transmission proteins, otherwise fuel consumption (which is too fast and localized) would be largely dissipated in the form of heat. For example, if ATP is hydrolyzed at a particular nucleotide-binding pocket within a motor, energy liberation from covalent bond breaking through phosphoryltransfer to water would dissipate as heat in a matter of picoseconds (30) and within a radius of a few Ångstroms (31). These are meaningless ranges considering the protein motions that take place in biological nanomachines, which are measured in milliseconds and tens to hundreds of Ångstroms (32, 33). It is thus the protein rearrangements, linked to binding/dissociating ATP/ADP, that efficiently transduce the energy potential within the adequate time and space scales.

**Figure 2:**
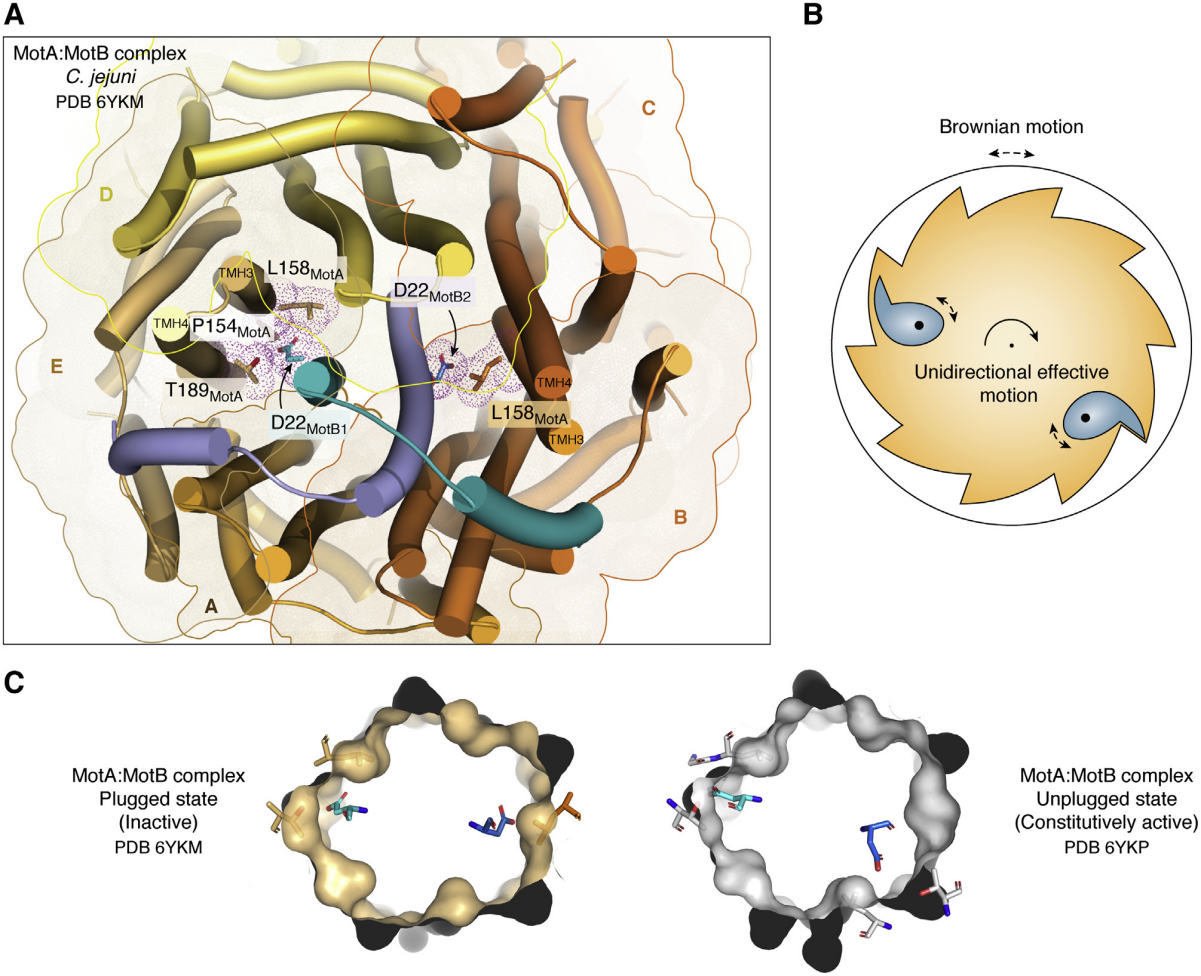
**The flagellar motor acts as a Brownian ratchet.***A*, cartoon depicting the cryo-EM structure of the stator MotA–MotB complex from *Campylobacter jejuni* in the “plugged” inactive state (PDB ID: 6YKM) (36). Toward the center, the MotB dimer is colored in *blue*, with different tones distinguishing the two monomers. The proton-transporter aspartate (D22) is labeled on each monomer and drawn as *sticks*. The pentamer of MotA chains, labeled from A to E, surrounds MotB. MotA monomers are distinguished in different tones of *yellow-to-orange*, and transparent surfaces with silhouettes also emphasize each MotA monomer’s position. MotA residues in contact with D22 are shown as *sticks*, together with their van der Waals surfaces displayed as *dots*. Note the distinct asymmetric environments that surround the proton transporter D22 in both MotB monomers. *B*, schematic drawing revisiting the mechanism of ratchet and pawl. Because of force applied to the pawl(s), they tend to remain close to the ratchet, whereas Brownian motion is induced in the entire system because of environmental thermal energy. The mechanism thus transduces the geometric asymmetry of the ratchet’s structure, into unidirectional rotation motion. *C*, simplified views of the MotA–MotB complex comparing two functional states, inactive (*left*) and active (*right*). The “plugged” inactive state is identical to *A* but simplified for clarity: MotB proteins are not shown except for the proton-carrier aspartates, and MotA is shown mostly as a molecular surface, with D22-contacting residues in *stick* representation. To the right, the constitutively active state—that is promoting proton transfer—was obtained by the authors using an “unplugged” N-term-truncated MotB mutant (PDB ID: 6YKP). Asymmetric features of MotA’s surface (“ratchet”) interfere with MotB’s aspartates (“pawls”) such that random motion of MotA only results in effective rotation in one direction. PDB, Protein Data Bank.

In the bacterial flagellar motor, ATP hydrolysis does not drive rotation. Instead, it is the regulated transport of protons or sodium cations along their electrochemical gradient, from the outside to the inside of the cell. Such proton-motive potential is transduced to mechanical work *via* allosteric conformational/dynamic rearrangements of key motor proteins (34). Recent cryo-EM data, obtained independently by two laboratories (35, 36), uncovered the molecular mechanism of such allosteric transitions, explaining with unprecedented detail how the energy is transduced through the stator to the rotor, forcing the flagellar motor to rotate. High-resolution 3D reconstructions of the stator piece of bacterial flagella were obtained by single-particle analysis (SPA). The stator complex comprises several units of two protein components, MotA and MotB (Fig. 1). For many years, the stator was thought to respect a 4:2 MotA:MotB stoichiometry (37, 38). However, those data were deduced from well-designed ^35^S-radiolabeling biochemical experiments, which however did not visualize the assembled protein complex directly. Now, cryo-EM clearly reveals an “asymmetric” 5:2 MotA:MotB ratio, a slight yet extremely relevant difference to previous interpretations (37). Furthermore, this 5:2 stoichiometry is conserved among many bacterial species, both Gram-negative (*Vibrio mimicus* and *Campylobacter jejuni*) and Gram-positive (*Clostridium sporogenes* and *Bacillus subtilis*), as well as across the entire family of MotA/MotB orthologs, such as PomA/PomB, the Ton transport ExbB/ExbD systems, and even the nonhomologous GldL/GldM gliding machines (35). The asymmetry within the stator assembly appears to be intimately related to the very capacity of exerting unidirectional mechanical work. The two MotB monomers lie side by side, constituting an inner core, which is surrounded by a slightly distorted pentagon of MotA helices (Fig. 2*A*). The 5:2 ratio constrains each MotB monomer to sit into nonequivalent environments at any given time: each MotB helix is forced to interact with a different constellation of MotA residues. It is precisely this helix that includes the key cation transporter amino acid within MotB, namely aspartate 22 (according to the *C. jejuni* numbering scheme), a strictly conserved residue in bacterial MotB orthologs across phyla (Fig. 2*A*). The dissimilar environments play a central role in allowing for a “see-saw” alternating motion, of the carboxylate-bearing Asp22 side chains.

The conformational change linked to the transport of each proton (a hydronium cation in water) is not very large, approximately 100-fold smaller than the estimated arc length traversed by the rotor (∼20–38 Å) per cation passage (36). This feature is more consistent with a biased diffusion mechanism, also known as a Brownian ratchet, rather than one based on a power stroke. In the latter, the magnitude of the protein shifts is typically on the order of several nanometers, that is, distances comparable to the dimension of the protein components themselves (31), with the swinging motion of myosin’s lever arm being a prototypical example (39). Directionally biased diffusion as a driver of mechanical movements (Fig. 2*B*), particularly as a means of powering a rotating motor device that produces effective work, received statistical thermodynamics support in the famous lecture “ratchet and pawl” by Richard Feynman (40). Essentially, the asymmetric configuration of the ratchet and a certain input of energy to fix the pawl(s) impose a unidirectional sense to the otherwise random Brownian motion. A simple ratchet can be illustrated by a round gear with asymmetric teeth and one or more pawls as pivoting spring-loaded fingers engaging the teeth (Fig. 2*B*). The energy instilled onto the pawl counteracts the random motion, and the asymmetric interaction with the ratchet is maintained, obstructing reversed rotation (Fig. 2*B*). The recent cryo-EM data (35, 36) indeed demonstrate that the Brownian ratchet mechanism is at play within the stator complex itself. Two features deserve special attention (Fig. 2*C*): (i) the force injected to the aspartic acid/aspartates (the pawls) on MotB is due to the binding of protons on one side, and their dissociation on the other, along the electrochemical gradient; the Asp carboxylate–bearing side chains rearrange spatially, according to them being charged or neutral, alternating such shifts in a see-saw fashion between both MotB monomers; and (ii) the molecular surface of the MotA pentamer (the ratchet) is irregular and offers an asymmetric junction to the moving aspartic acid/aspartates. It is thus clear that MotA and MotB constitute a fully functional motor on their own. MotA acts as the moving rotor, whereas MotB is the stator, fixed to a static reference such as the PG through its C-terminal PG-binding OmpA domain.

The interaction of the pentamer of MotA with the FliG multimer that constitutes the C ring allows the stator to transmit the rotary motion to the flagellar rotor (Fig. 1). *In situ* images of entire motors embedded within the inner membrane of *Borrelia burgdorferi* and *Vibrio alginolyticus* were recently obtained by cryo-ET at ∼20 Å resolution (41, 42), demonstrating that MotA and FliG act as two coupled cogwheels. This mechanism allows to switch the sense of rotation of one of the cogwheels by inverting the direction of coupling between the two. While MotA always rotates clockwise (CW), FliG on the C ring can turn either CW or counter-clockwise (CCW). Flipping the FliG–MotA interface is enacted by a conformational rearrangement of the C-terminal domain of FliG, according to the states that the FliG-bound FliM–FliN switch complex adopts in response to stimuli (Fig. 1). Not only do the shape and molecular details of the purified stator complex (35, 36) fit consistently into the *in situ* cryo-ET volumes of the whole motor (41, 42), but the latter images also lend strong support to the flipping mechanism and hence to the two-cogwheel gear model. The tomographic volumes were reconstructed from motors locked in the CW or CCW states, and the comparison of both uncovers a large conformational change of the C ring, whereas the stator positions remain unchanged. In the case of *B. burgdorferi* (42), the wall of the C ring closes its upper membrane-facing diameter relative to the motor axis to ∼55 nm in the CCW state, whereas opening it to ∼62 nm in the CW state. The same pattern of open/closure rearrangements also takes place in other species (41). On the other hand, several MotA subunits are organized into a conical tube at invariable positions with respect to the motor axis. In the CCW state, the C ring structure exhibits its FliG subunits interacting with the portion of MotA that is closer to the motor axis. The switch to the CW state forces the FliG moieties to interact with the opposite part of the MotA tube, located farther from the motor axis. Overall, the two cogwheels coupled-flipping mechanism constitutes a clever evolutionary solution to the problem of rotational switching in bacteria flagella.

**Figure 3:**
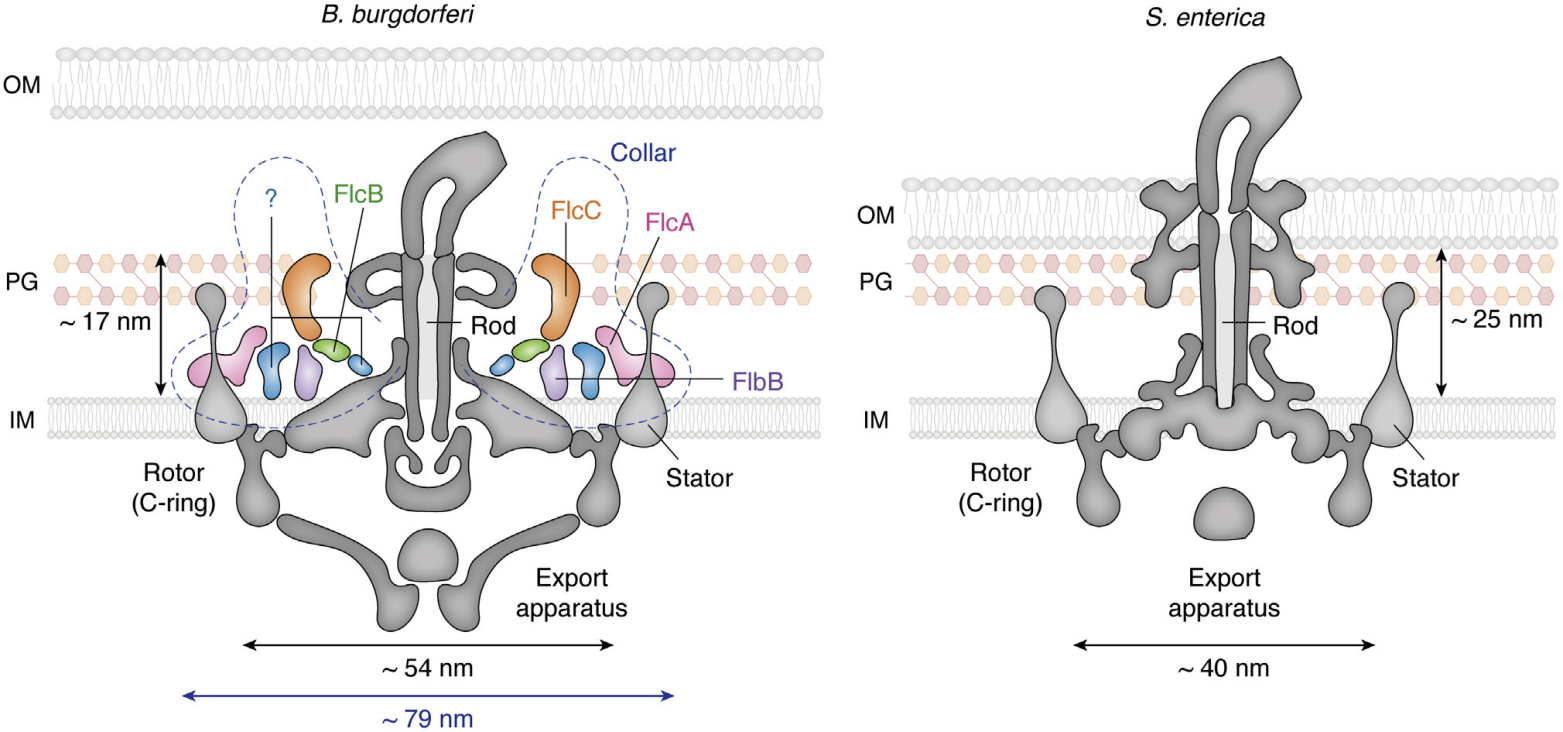
**Basal bodies of *Borrelia burgdorferi* (endoflagellum) and *Salmonella enterica* (exoflagellum).** Schematic illustrations highlighting the positions of different components of the basal body, drawn to scale for comparative purposes, based on currently available *in situ* cryo-electron tomographic data. Note the presence of the collar: a large, 79 nm wide, protein complex unique to Spirochetal endoflagella, with identified protein subunits indicated and additional components that remain to be localized. The rod is shorter in endoflagellates (17 nm in *B. burgdorferi versus* 25 nm in *S. enterica*), but the motor has a larger radius (54 nm *versus* 40 nm), key to generate the high torques observed in spirochetal endoflagella. IM, inner membrane; OM, outer membrane; PG, peptidoglycan.

Different bacteria exhibit a wide range of torques as measured on their rotating motors (26). Recalling that torque depends not only on the force applied but also on the radius of the rotating object, evolution indeed showcases different means by which flagellar motors achieve higher torques. As a first example, the maximum number of stator complexes recruited by the rotors from different species has been observed to vary from 11 to 17 (26, 43), modulating the total force applied for rotation. A second way of increasing torque has also been observed, namely by increasing the motors’ radii (26), and both ways are often selected simultaneously. Altogether, higher torques correlate with higher swimming speeds and greater ability of bacteria to move through viscous media. Varying torque magnitudes reflect adaptations to disparate lifestyles, ranging from ∼350 pN·nm in *Caulobacter crescentus* (44), ∼2000 pN·nm in *Salmonella* and *E. coli* (45, 46), all the way to higher values in species adapted to swim in viscous media, such as *Helicobacter pylori*, which displays torques of ∼3600 pN·nm (47). Remarkably, Spirochetes can go higher, up to >4000 pN·nm of torque, among the highest reported for any bacteria (48). Consistent with this, the flagellar basal bodies from Spirochetes also stand out among the widest (26) (Fig. 3), enabling them with unique abilities to drill through tissues and rapidly disseminate within infected hosts with extreme efficacy. Such high-torque motors influence the singular organization of the different portions of spirochetal flagella, including the hook and the filament (see further details later). Likely related to such intense rotation, the spirochetal motors have evolved an entirely novel element within the periplasmic portion of the basal body known as the collar (49, 50) (Fig. 3). The collar is a multiprotein complex, essential for normal flagellar biogenesis and motility (51, 52, 53). Its unique structure increases the basal body’s structural stability and is involved in recruiting 16 stator units per motor (51). Five collar protein components have been identified so far, four of which have been localized unambiguously in *in situ* cryo-ET images (Fig. 3): FlbB (the base), BB0236 (interacts with FlbB and is involved in the collar’s assembly), FlcA (forms an outer turbine-shaped structure interacting directly with stators), FlcB (contributes to the middle portion of the collar), and FlcC (is directly involved in collar formation and stator assembly). Among these, BB0236 (52) was thought to occupy the position that has later been assigned to FlcB (51), leaving open the question as to its actual localization. Still, unaccounted density is observed between FlbB and FlcA (Fig. 3), which could correspond to BB0236 and be consistent with genetic and phenotypic evidence. Beyond these five proteins, extra volume exists in this large assembly, and future research will allow the full elucidation of its components and the complete sequence of events leading to its biogenesis.

## The flagellar hook: Two states *versus* multiple conformations of its protomers

**Figure 4:**
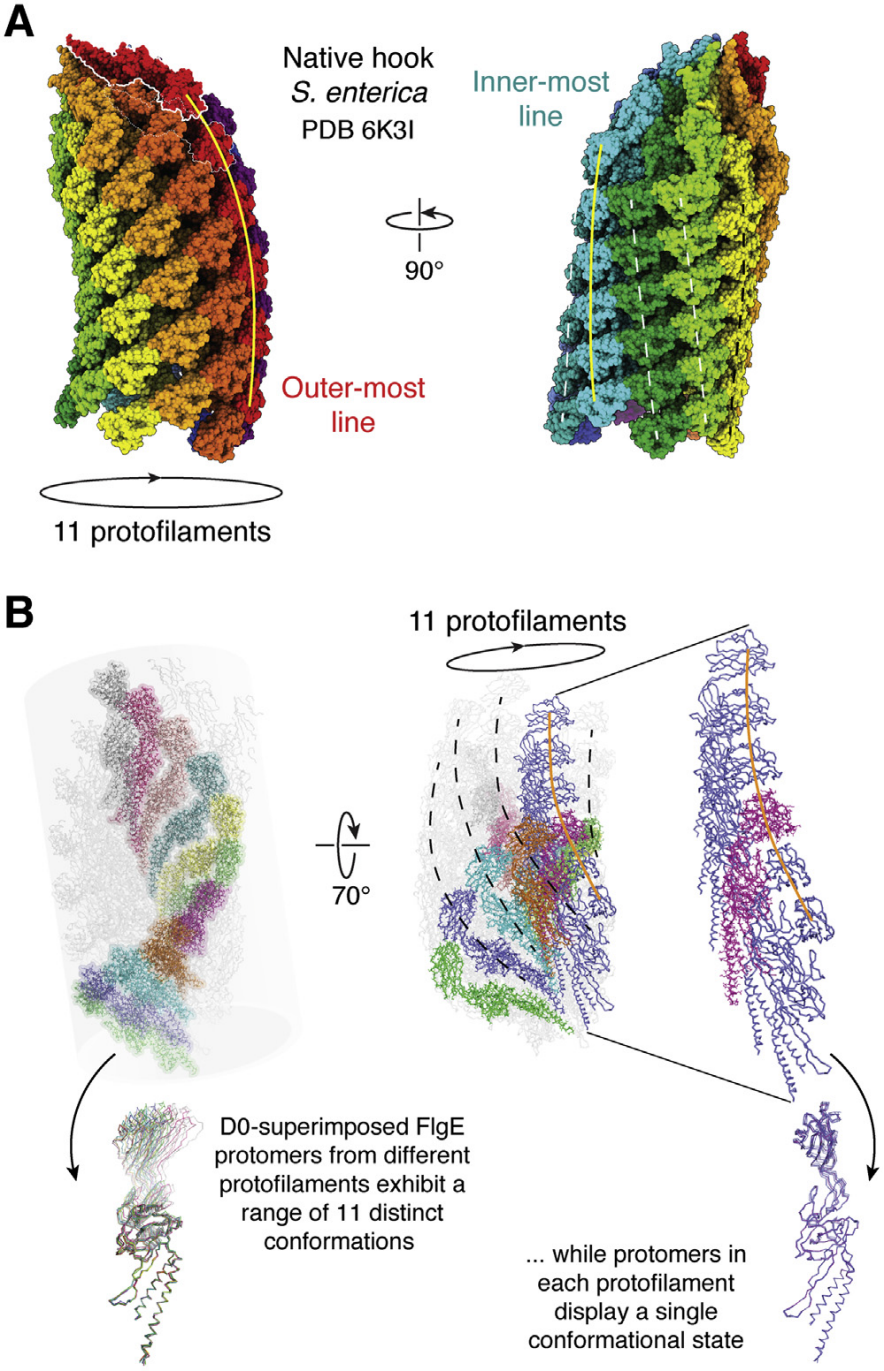
**FlgE protomers in the flagellar hook adopt a continuous array of conformations, one for each protofilament.***A*, 3D structure of the native hook from *Salmonella enterica* (PDB ID: 6K3I), viewed from two orthogonal perspectives. Each of the 11 protofilaments are distinguished with different colors, and the natural curvature of the assembly allows to identify outermost (convex surface) and innermost (concave surface) lines. FlgE protomers are most separated among them along the former and most tightly packed along the latter line. *B*, each protofilament exhibits a distinct conformation, contradicting a simpler two-state conformational rearrangement switch for flagellin-like protomers. To the *left of the panel*, one FlgE protomer from each of the 11 protofilaments is highlighted in strong colors, and to the *bottom*, they are superposed, showing a substantial and continuous rearrangement among them. By changing the perspective to the *right*, one protofilament is chosen (marked with an *orange line* through its trajectory), zooming in to confirm that all protomers within the protofilament are nearly identical in conformation. Note that the conformational rearrangement of FlgE protomers is highly cooperative (consolidating each protofilament) and happens fast and synchronously as the hook rotates. PDB, Protein Data Bank.

The hook is a ∼55 nm long, typically curved tubular structure of ∼120 protomers of the protein FlgE. The protomers arrange according to a helical symmetry, resulting in a supramolecular assembly that is composed of 11 protofilaments packed side by side, with each protofilament following a helical trajectory along the long axis of the hook (Fig. 4*A*). The hook functions as a universal joint (Fig. 1), allowing torque transmission from the rotating motor to the filament, despite these two structures not being coaxial (54). FlgE proteins from different species adopt a conserved 3D fold in spite of fairly large sequence disparities (55, 56, 57). The structure of FlgE comprises a minimum of three domains: D0, which forms the innermost core of the tubular structure, and domains D1 and D2 successively constituting concentric outer shells of the tube (58). These three domains are highly conserved and may encompass the entire FlgE polypeptide in some species (*e.g.*, *S. enterica*). In other organisms, such as ε-proteobacteria (*e.g.*, *C. jejuni* and *H. pylori*), two additional domains are found, D3 and D4, significantly extending the length of more external portion of FlgE (55, 56). Also, a long “L-shaped” β-hairpin within domain D0 (known as the “L-stretch”) connects domains D0 and D1 in *C. jejuni* FlgE. The extra contacts because of a longer protomer, together with the L-stretch structure, make *C. jejuni*’s hooks stronger and stiffer compared with those from Enterobacteria, while maintaining the capacity to curve. These are adaptive features underlying the naturally efficient motility of *C. jejuni* in viscous environments (56), which require flagella rotating at higher torque regimes. Indeed, in *S. enterica*, there is a β-hairpin equivalent to the L-stretch contacting domains D0 and D1, known as domain Dc. However, it is considerably shorter than its *C. jejuni* counterpart (59).

The molecular interactions among FlgE protomers, and their implication for the hook’s universal joint function, have been the subject of many studies over the last 2 decades. Particularly intriguing, when bacteria swim, the hook is strongly curved at a fixed angle, producing defined convex and concave sides of the assembly that remain bent in this direction with respect to the cell body while rotating. However asymmetric, this bending is enforced by an ensemble of otherwise chemically identical FlgE protomers, raising the question of how this is achieved. Using molecular dynamics simulations, the distinct bending flexibilities of differently located protomers was proposed to result from differential compression and extension of FlgE intersubunit distances, when comparing neighboring protofilaments among them (54, 58). To undergo such conformational changes with low energy costs, mechanisms involving sliding between subunits at the intersubunit interface, rearrangements of hydrogen bonding pairs, and relative domain motions between domains D1 and D2 were proposed (58). Studies of the flagellar filament, an 11-protofilament structure homologous to the hook (see later) in which flagellin protomers adopt the same fold as FlgE, revealed that the subunit protomers adopt one of two possible conformations (60). Known as L and R (for the handedness of the helical lattice they induce when assembled), these two conformational states of flagellin protomers provided an explanation for the diverse supercoiling forms and functional properties of the flagellar filament. According to the combination of L and R protofilaments in different ratios within the supramolecular assembly, a two-state conformational rearrangement mechanism became the paradigmatic model (61), and a similar mechanism was thought to operate in controlling the hook’s shape and flexibility (62). However, this hypothesis has recently been contradicted. Two groups independently determined the structure of the hook from *S. enterica* in its native curved state by cryo-EM SPA at 2.9 Å (63) and 3.6 Å (64) resolution, without imposing any symmetry. They both validate the initial compression/extension mechanism underlying the hook’s flexibility and curvature. However, they show that the FlgE subunits in the hook do not respond to a two-state conformational switch, instead populating a continuous ensemble of conformations, each one of them cooperatively propagated along the individual protofilaments (Fig. 4*B*). This notable observation raises the possibility that similar continuously flexible configurations could also be at play in the flagellar filament, for which more studies of native naturally curved filaments will be relevant. The change of paradigm, from a simpler two-state model to a continuous array of protomer conformations, is the result of technical improvements over the last several years in the field of cryo-EM image analyses. The initial hypothesis received support from observations using mutated variants of flagellin. Such mutants resulted in conformationally homogenous filament preparations that were better suited for high-resolution cryo-EM studies (65, 66). Recent methodological developments paved the way to studying hooks in their native supercoiled states, allowing observations of the hook protomers’ flexibility coupled to allosteric cooperativity, revealing how FlgE subunits expand and compress, enabling the hook to act as a universal joint (Fig. 4*B*). However, the molecular basis of the defined asymmetric bending is still a puzzling issue. Each protofilament is composed of similarly configured FlgE protomers such that, as the assembly rotates, each protofilament will rapidly traverse the other 10 different conformations: what constrains each position among the different longitudinal directions of the assembly to adopt a unique conformation? That each protofilament position of the curved rotating hook only lodges a particular FlgE conformation, appears to imply that external forces—perhaps the connection to the rotating basal body’s rod—impose some degree of asymmetry, subsequently fixed and amplified *via* inter-FlgE cooperativity. Indeed, recent observations of temporary “hook locking” during CCW to CW motor reversal in *Salmonella* and *E. coli* (67) uncover initial hints that compression/expansion of protofilaments might actually be controlled. In cell reorientation events, rapid and transient hook locking fixes the concave and convex faces of the hook during rotation, with the structure no longer acting as a universal joint but instead implying that each protofilament has been fixed in a unique conformation (68).

**Figure 5:**
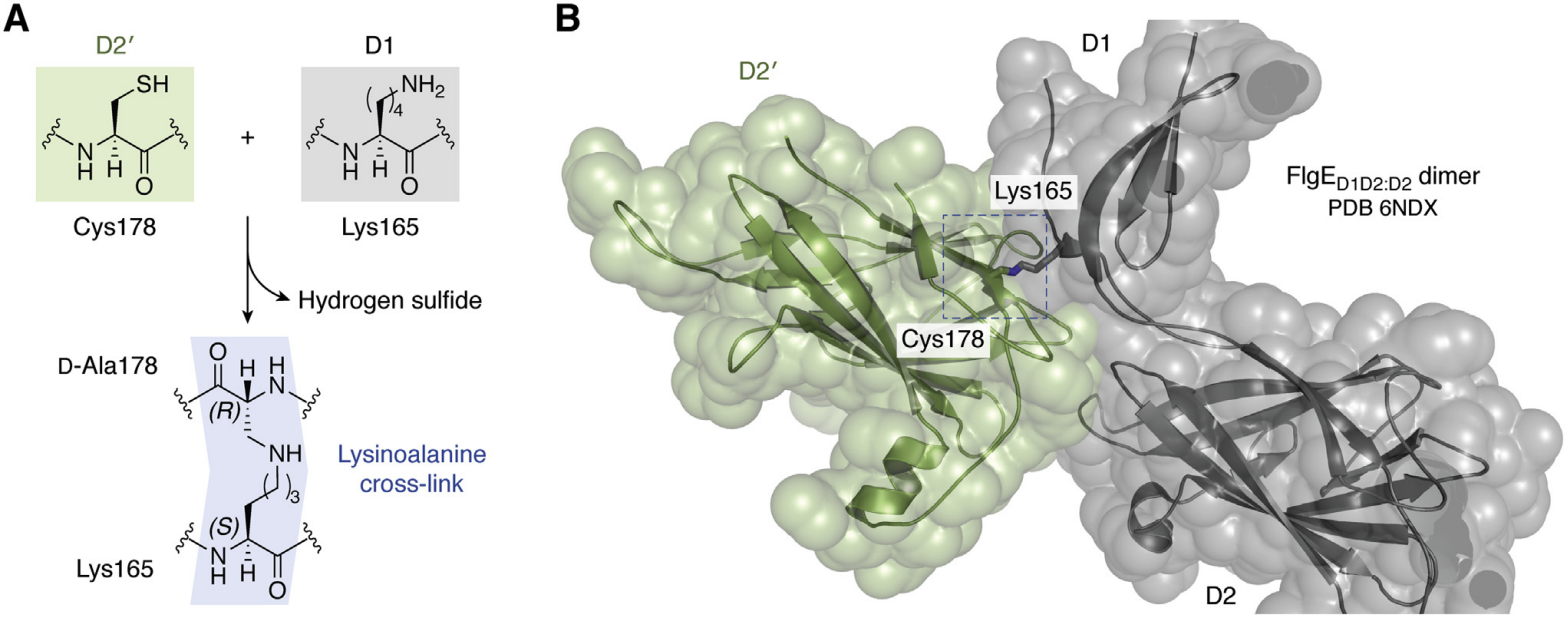
**FlgE protomers in Spirochetes are covalently crosslinked in the assembled hook.***A*, autocatalytic cross-linking reaction at the D1–D2′ domain interface, generating a lysinoalanine covalent adduct. The reaction occurs between D1 residue Lys165 and Cys178 on the D2′ domain of an adjacent FlgE subunit in the assembly. The reaction involves three distinct biochemical steps: oligomerization of FlgE subunits *via* D0 interactions, β-elimination of the Cys178 thiol with the release of hydrogen sulfide as a byproduct, and aza-Michael addition of Lys165 from an adjacent FlgE monomer to yield lysinoalanine. *B*, structure of the resulting crosslinked FlgE_D1D2:D2_ dimer (PDB ID: 6NDX). Note the lysinoalanine residue (*blue dashed box*) located at the interface between the D2 domain of one FlgE protomer (*green*) and the beginning of domain D1 of a neighboring protomer (*gray*), near the linker segment between domains D1 and D2. PDB, Protein Data Bank.

The sequence diversity of FlgE proteins in different bacterial phyla suggests that hooks have diverged to specifically fit distinct motility requirements (56). When γ-proteobacteria like *V. alginolyticus* reverse their swimming direction, the hook of their single polar flagellum is homogenously compressed until it buckles, producing a kink between the filamentous appendage and the cell body. In this configuration, the hook stops acting as a universal joint and instead drives filament gyration pushing the cell sideways in a flicking motility mode (69). On the other hand, ε-proteobacteria and Spirochetes illustrate adaptive variations to swim efficiently in highly viscous media. Such an ability is essential for host colonization and virulence in pathogenic species from these bacterial taxa, enabling them to cross gastrointestinal mucous and tissues, respectively. Some of their flagellar adaptations include specific modifications of their hooks, allowing them to bear with their higher torque motors (see previous one). As mentioned earlier, FlgE protomers from *C. jejuni* include the extra domains D3 and D4, which introduce additional contacts within and between protofilaments, making their hooks stiffer (56). Even more remarkably, it was recently shown that spirochetal FlgE proteins evolved an enzymatic activity that self-catalyzes an interpeptide crosslinking reaction (70), forming an unusual intersubunit lysinoalanine covalent adduct, with no need of other enzymes or cofactors (Fig. 5*A*). The crosslinking site sits at the D1–D2 domain interface, whereby D1 residue Lys165 reacts with Cys178 on the D2 domain of an adjacent FlgE protomer, both residues being close to each other in the assembled hook (Fig. 5*B*). Such covalent bonding eventually results in the crosslinking of all FlgE protomers in the hook (70, 71). This reaction was observed in different spirochetal genera (72), all of which possess periplasmic flagella. The covalent polymerization of the entire hook provides them with great mechanical stability, consistent with spirochetal motors producing the highest torques among bacterial flagella (26), necessary to drill through highly viscous extracytoplasmic matrix and tissues.

In sum, the chemically identical protein subunits that form the hook have adapted their structure to undergo precise conformational shifts that depend on physical interactions with neighboring subunits and flagellar structures. Variations in the protomers’ sequences further expanded a number of functional adaptations, which efficiently support increasing workloads and correlated torques. Thus, flagellar hooks exhibit an astonishing combination of structural stability and flexibility, promoting efficient torque transmission, while enabling bacteria to move through widely diverse media.

## The flagellar filament: Not always a helically symmetric homopolymer

The filament has been extensively studied in many Gram-negative and Gram-positive bacteria (13, 73). It is an extremely long homopolymeric assembly of flagellin protomers packed in a helical array, very similarly organized as the 11-protofilament structure of the hook. Besides serving as a propeller for translational motility, the filament is involved in additional functions, such as adhesion, pathogenicity, and immunomodulation (12, 74). The first high-resolution 3D reconstruction of an assembled filament was obtained by cryo-EM SPA (66). A point-mutated form of the flagellin FliC from *S. enterica* was used as a means of avoiding natural supercoiling, by stabilizing assemblies that contain only right-handed (R) protofilaments. Such straight filaments dramatically improved cryo-EM resolution, a breakthrough for bacterial flagellar structural biology. Since then, many other filament structures have been solved, confirming a well-conserved organization with 11 flagellin protofilaments positioned around a central ∼2 nm-wide channel. Flagellin protomers are secreted by the flagellar secretion apparatus and reach the distal growing tip of the filament through the central channel (75), where they are chaperoned to fold in place by FliD or filament-capping protein (76) (Fig. 1). Originally identified as hook-associated protein HAP2, FliD is secreted early on, binds to the tip of the hook, and assists in filament initiation. It then remains attached during the whole filament polymerization process, promoting flagellin self-assembly and avoiding flagellin leakage (77, 78). While early observations led to the paradigm that FliD exerts its filament-capping function as a pentamer (79), it has also been reported to function as a species-specific oligomer, including tetrameric, pentameric, or even hexameric variants (80, 81).

**Figure 6:**
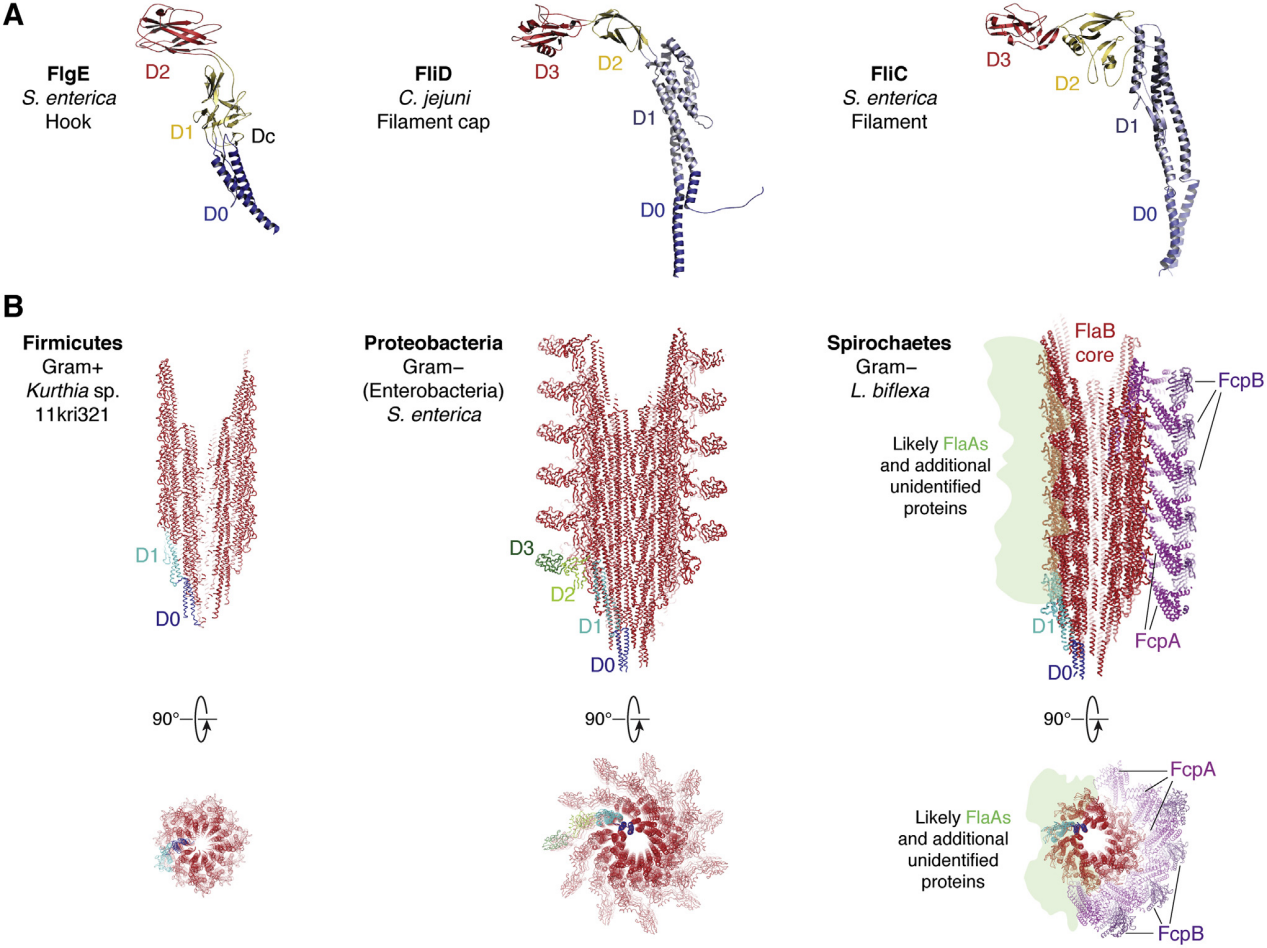
**The flagellar filament.***A*, the protomers of the hook (FlgE), the filament capping complex (FliD), and the filament core itself (FliC flagellin) are shown as cartoons, with domains highlighted in different colors. Note that the three share a similar 3D architecture and domain organization (D0 to D3 as indicated), defining a flagellin-like fold. *B*, evolutionary constraints have selected filaments with increasing and variable complexity. A flagellin core comprising the two all-helical domains D0 and D1 is always present. *Leftmost panel*, a bare D0–D1 core composes the native filaments of, for example, Firmicutes including *Bacillus subtilis* (the filament from the related Firmicute *Kurthia* sp. is shown; PDB ID: 6T17). *Center panel*, in Enterobacteria, the flagellin protomers have two extra domains, D2 and D3, decorating the core and protruding as spikes from it, such as in *Salmonella enterica* (PDB ID: 1UCU, applying the reported helical symmetry to generate the ensemble). *Rightmost panel*, a case of extreme complexity is observed in Spirochetes, where a D0–D1 flagellin core is sheathed asymmetrically by several different protein species on either side of the curved appendage, illustrated by *Leptospira biflexa* (PDB ID: 6PWB; the sheath protein FlaA, and likely additional unannotated proteins, is not localized with certainty, yet preliminary data support their indicated position). PDB, Protein Data Bank.

The fully assembled filament is a robust protein polymer that rotates fast, propelling the entire bacterial body at great speeds. The most accepted paradigm about the filament’s architecture is that of a radially symmetric tube, built by polymerization of flagellin as a single protein species (FliC or paralogous/orthologous isoforms of it). Flagellin protomers are arranged according to a simple helical symmetry, that is, a defined helical radius, with a precise rise (Å) and twist (º) per consecutive FliC protomer. The FliC protomer shares a very similar structure to the hook FlgE and to the cap protein FliD (Fig. 6*A*). The N- and C-terminal regions of FliC comprise ∼250 amino acids each, folding together into two all-helical domains named D0 and D1, each incorporating portions from both terminal sequences (82). D0 and D1 are both buried concentrically within the core of the assembled filament, being responsible for polymerization with neighboring protomers (73, 83). The inner D0 ring delimits a central 2 nm-wide channel that is continuous with the one present along the basal body’s rod and the hook (Fig. 6*B*, *leftmost panel*). Often, in between the highly conserved D0 and D1 sequence segments, sits a stretch of sequence of variable length, which encodes surface-exposed domains, radiating as spikes from the central D0–D1 core (Fig. 6*B*, *central panel*). Such additional domains are frequently two, globular and β-sheet–containing (named D2 and D3), which typically include more variable sequences that provide interspecies diversity (84), and can even harbor enzymatic activities (85). Even more than two intervening domains may be present (86), all the way to the recently identified giant flagellins (87, 88), some of which surpass 1000 amino acids in length (compared with the 495 residues of *S. enterica* FliC). On the other hand, it is also well known that many species only possess the conserved core D0–D1 structure within their flagellins, such as in most Firmicutes, including *B. subtilis* (73), or in Spirochetes, in which the FliC ortholog is known as FlaB (Fig. 6*B*, *rightmost panel*). Moreover, while *B. burgdorferi* possesses a homopolymeric FlaB core (89), most Spirochete species have several flagellin paralogs or isoforms (90, 91), with *Leptospira* spp. simultaneously expressing four of them (92). Concerning their distribution throughout the filament, it is not yet known whether these different flagellin isoforms are evenly spread or instead restricted to defined subregions.

The widely accepted paradigm of a symmetric homopolymeric filament acting as an extracellular freely rotating whip has been challenged with the progressive identification of more complex assemblies. Especially illustrative is the case of Spirochetes, which possess more than one protein component in their unique endoflagella, located within the periplasmic volume between the two cell membranes (93). Notably, the FlaB assembly forms a flagellin core that is coated with a proteinaceous sheath (89, 90, 94, 95), adding complexity and allowing for larger interspecies variability (Fig. 6*B*, *rightmost panel*). The universal component of the filament sheath in Spirochetes is FlaA, which has no homology to FlaB and can be present in one or more isoforms. FlaA proteins include a signal peptide, strongly suggesting that they are secreted into the periplasm *via* the classic Sec-dependent pathway, and thereafter recruited onto the filament (96, 97). The function of FlaA is not completely understood, but Δ*flaA* knockout mutants present motility defects (93, 97, 98), and normal filament assembly can be severely impaired (99). Besides FlaAs, additional protein species can be recruited onto the filament sheath of several Spirochetes (94, 99, 100, 101, 102, 103). Improvements in SPA cryo-EM and cryo-ET with subtomogram averaging analyses have recently provided the first 3D structures of complete filaments from wildtype *Leptospira* spp. and selected sheath protein mutants, reaching near-atomic resolution (94, 99). The flagellar filament from *Leptospira* is currently the most complex to be reported in terms of protein composition and architecture (Fig. 6*B*, *rightmost panel*). The cryo-EM data of *Leptospira* filaments combined with X-ray crystallographic analyses of individual flagellar proteins (94, 100) uncovered a markedly curved filament, possessing a helically symmetric 11-protofilament core, which contains four FlaB flagellin isoforms (92). This core is surrounded by a strongly asymmetric sheath (94) (Fig. 6*B*, *rightmost panel*), composed of two FlaA isoforms (FlaA1 and FlaA2) (98), plus several additional constituents unique to the *Leptospira* genus: (i) flagellar coiling protein A (FcpA), one of the most abundant components of *Leptospira* endoflagella and essential for motility and pathogenicity (92, 100, 101) and (ii) flagellar coiling protein B (FcpB), also required for normal motility and cell morphology (102). The sheath asymmetry is revealed by the presence of distinct protein species on the convex and concave sides of the assembly (94, 99), in stark contrast with the symmetric filaments of exoflagellated bacteria (Fig. 6*B*). The convex, or outer side of the sheath, is composed of a two-tiered polymer, with FcpA protomers organized as an inner layer directly contacting the FlaB core, and a surface-exposed layer of FcpB moieties largely covering the FcpA sheet. The intricate network of intermolecular contacts, exerting asymmetric forces onto the FlaB core, elucidate the molecular basis of filament stiffness and supercoiled curvature (94, 99) that are essential for *Leptospira* motility. The fact that FcpA contacts the core directly and interfaces with the external layer of FcpB is consistent with a sequential recruitment of both proteins and with a stronger phenotypic effect observed in the Δ*fcpA* mutant strain (which loses both Fcp proteins) compared with Δ*fcpB* (102). The concave inner side of the filament displays a completely different protein composition (94), with densities that could be assigned to FlaA2 and to a novel protein that had not been annotated so far (99). Extra EM densities could not yet be assigned to known proteins and likely correspond to FlaA1 and other hypothetical proteins.

Taking all the evidence together, phylogenetic variations of bacterial flagella, most likely related to environmental adaptations, can lead to filament structures that lose their characteristic symmetry, adding a richer complexity to the system.

## Final remarks and future trends

The last 3 years have seen major scientific breakthroughs that changed the way we understand bacterial locomotion. *In situ* images of entire motors embedded within the inner membrane of bacterial cells have been obtained by cryo-ET (41, 42), and near-atomic resolution images of MotA–MotB stator complexes from several bacterial species have been obtained by cryo-EM SPA (35, 36). These data strongly suggest that evolution has shaped flagellar stator proteins to move as a Brownian ratchet device of nanometric dimensions, ruling out alternative proposals of perhaps more intuitive power-stroke machines (104). A gear mechanism is also introduced as the molecular means by which the rotating stator transmits its motion to the rotor. Acting as two coupled cogwheels, MotA and FliG form a gear that ultimately produces the motor’s torque. This gear turns out to be a clever solution to the problem of rotation direction switching because the ratchet and pawl device has a single direction of rotation intrinsically imprinted in its structural geometry (Fig. 2*B*). The two-cogwheel gear allows the sense of rotation of one of the cogwheels (the C ring of the rotor on the base of the flagellum) to switch by simple inversion of the direction of coupling between the two. More precisely in the case of the motor, this switching device corresponds to the interface between MotA (which always rotates CW) and FliG on the C ring (known to turn either CW or CCW). Flipping the FliG–MotA interface is enacted by a conformational rearrangement of the C-terminal domain of FliG (41, 42), according to the states that the FliG-bound FliM–FliN switch complex adopts (Fig. 1). It has been pointed out that high-resolution structures of the C ring–stator complex, in both CW and CCW states, are still needed to fully understand the interactions between MotA and FliG (105), as it is this latter interface that ultimately underlies the generation of torque for the entire motor.

Despite the huge progress in understanding bacterial locomotion, there is still a relatively larger knowledge gap concerning Gram-positive species. Seminal studies have greatly contributed to uncovering details of the *B. subtilis* flagella (73), more recently including the switch complex (106). Although the critical components for rotation are organized similarly to *E. coli*, *B. subtilis* possesses a larger protein FliY in the switch complex, instead of FliN, and actually the switch of its rotational movement responds oppositely to chemotactic phospho-CheY signaling (106). Future structural studies will certainly shed light onto distinct rotational switch regulation mechanisms among different bacterial species.

Intriguing questions remain open about the workings of bacterial flagella and their diversity in different taxa. Concerning the motor, several of its protein complex components rotate very fast, while interacting directly with the lipid bilayer of the cell membrane. How are friction and mechanical stress handled to maintain membrane integrity? This issue directly concerns structures like the MS ring and the MotA surface of the stator subunits, both of which comprise large portions fully embedded in the membrane. While in Gram-negative organisms, the axial rod is surrounded by bearings that isolate its rotation from the PG and the outer membrane (*via* the P and L rings, respectively), there is no equivalent bearing to envelop the inner-membrane motor components (Fig. 1). Constituted by 34 protomers of FliF, the MS ring associates tightly to the cytoplasmic rotor (C ring) *via* FliF–FliG interactions (107), while covering the proximal surface of the axial rod across the periplasm (108). The MS ring is thus bound to rotate consolidated with the C ring and the axial rod. Fast-turning proteins could destabilize the fragile membrane structure, yet the mechanisms to avoid it remain enigmatic. Also puzzling is the molecular basis of the hook’s asymmetric bending (59, 64). How is the structural asymmetry maintained as the assembly rotates, considering that each protofilament is composed of similarly configured FlgE protomers? (63) *In situ* single-molecule approaches combining light microscopy and EM will likely shed light on these phenomena in the near future.

Flagellar diversity during evolution makes the Spirochetes a most fascinating model. Several recent accomplishments studying their unique endoflagella show how the same structural elements can design a radically different locomotion mechanism in bacteria. Endoflagella share the same general organization as exoflagella, yet the former possess no extracellular portions, so that the long filaments cannot work as free beating propellers to produce thrust and push the cell body. In a way that is not yet fully understood, Spirochetes use the flagellar machinery to drag their own cell body. Taking advantage of the sinusoidal, or even bluntly spiraled shape of the cell itself, the effected rotation produces a screw motion of the cell body and consequent forward thrust (24). The periplasmic space is quite limited, constraining the hook and filament to be very close or in contact to other cell components (109), such as the PG and the outer membrane and its proteins, especially ones that protrude into the periplasm from the inner leaflet. This framework for locomotion is very likely to impose higher workloads to the flagellar motor, which needs to produce considerably more torque to drag the entire cell (46, 48). Larger motors (Fig. 3), equipped with additional structures such as the collar (to maintain the basal body’s stability), are coherent with several other adaptations in each section of the appendage, including the extensive covalent crosslinking of the hook’s protomers (Fig. 5) and the presence of a complex proteinaceous sheath covering the filaments’ core (Fig. 6*B*). The strong asymmetry in the organization of the filament sheath, observed for the first time in *Leptospira* (94, 99), is striking and still enigmatic as to how it impacts on the endoflagellar-driven motility mechanism. The sheath’s asymmetry enforces essential stiffness and curvature properties of the endoflagellar filament and manifests both in the heterogenous distribution of protein species on either side of the curved assembly as well as in the mismatch with the 11-fold helical symmetry of the flagellin core. Considering that the sheath proteins are likely recruited from the periplasm onto the extant core, an open question remains as to whether the asymmetry arises because of extrinsic factors within the confined volume of the periplasm or because of intrinsic asymmetry within the flagellin core, possibly because of a patchy distribution of different FlaB isoforms. Future research combining genetics and structural biology approaches will allow the elucidation of the mechanisms of this new way of swimming motility.

The impressive technological progress in the field of structural biology, which is now merging with advanced bioimaging techniques, provides us with powerful tools to uncover the detailed mechanisms of bacterial locomotion. The relevant questions that remain unanswered and the captivating ways that different bacteria employ to swim in different environments guarantee an exciting research journey ahead.

## Conflict of interest

The authors declare that they have no conflicts of interest with the contents of this article.
